# Prothrombotic Genetic Mutations Are Associated with Sub-Clinical Placental Vascular Lesions: A Histopathological and Morphometric Study

**DOI:** 10.3390/cimb47080612

**Published:** 2025-08-04

**Authors:** Viorela-Romina Murvai, Anca Huniadi, Radu Galiș, Gelu Florin Murvai, Timea Claudia Ghitea, Alexandra-Alina Vesa, Ioana Cristina Rotar

**Affiliations:** 1 Doctoral School of Biological and Biomedical Sciences, University of Oradea, 1 University Street, 410087 Oradea, Romania; rominna.cuc@gmail.com (V.-R.M.);; 2 Department of Obstetrics and Gynecology, Emergency County Hospital Bihor, 65 Gheorghe Doja Street, 410169 Oradea, Romania; 3 Department of Surgical Sciences, Obstetrics and Gynecology, Faculty of Medicine and Pharmacy, University of Oradea, 1 University Street, 410087 Oradea, Romania; ancahuniadi@gmail.com; 4 Department of Neonatology, Faculty of Medicine and Pharmacy, University of Oradea, 1 University Street, 410087 Oradea, Romania; raduoradea@yahoo.co.uk; 5 Department of Neonatology, Emergency County Hospital Bihor, 65 Gheorghe Doja Street, 41069 Oradea, Romania; 6 Surgery Department, Faculty of Medicine and Pharmacy, University of Oradea, 1 University Street, 410087 Oradea, Romania; gelu.f.murvai@gmail.com; 7 Pharmacy Department, Faculty of Medicine and Pharmacy, University of Oradea, 1 University Street, 410087 Oradea, Romania; 8 1st Department of Obstetrics and Gynecology, “Iuliu Haţieganu” University of Medicine and Pharmacy Cluj-Napoca, 400012 Cluj-Napoca, Romania; cristina.rotar@umfcluj.ro; 9 1st Clinics of Obstetrics and Gynecology Cluj-Napoca, Emergency County Clinical Hospital Cluj-Napoca, 400006 Cluj-Napoca, Romania

**Keywords:** thrombophilia, placental pathology, genetic mutations, factor V Leiden, MTHFR polymorphism

## Abstract

Background: Inherited thrombophilia is increasingly recognized as a contributing factor to placental vascular pathology and adverse pregnancy outcomes. While the clinical implications are well-established, fewer studies have systematically explored the histopathological changes associated with specific genetic mutations in thrombophilic pregnancies. Materials and Methods: This retrospective observational study included two cohorts of placental samples collected between September 2020 and September 2024 at a tertiary maternity hospital. Group 1 included women diagnosed with hereditary thrombophilia, and Group 2 served as controls without known maternal pathology. Placentas were examined macroscopically and histologically, with pathologists blinded to group allocation. Histological lesions were classified according to the Amsterdam Consensus and quantified using a composite score (0–5) based on five key vascular features. Results: Placental lesions associated with maternal vascular malperfusion—including infarctions, intervillous thrombosis, stromal fibrosis, villous stasis, and acute atherosis—were significantly more frequent in the thrombophilia group (*p* < 0.05 for most lesions). A combination of well-established thrombophilic mutations (Factor V Leiden, Prothrombin G20210A) and other genetic polymorphisms with uncertain clinical relevance (MTHFR C677T, PAI-1 4G/4G) showed moderate-to-strong correlations with histopathological markers of placental vascular injury. A composite histological score ≥3 was significantly associated with thrombophilia (*p* < 0.001). Umbilical cord abnormalities, particularly altered coiling and hypertwisting, were also more prevalent in thrombophilic cases. Conclusions: Thrombophilia is associated with distinct and quantifiable placental vascular lesions, even in pregnancies without overt clinical complications. The use of a histological scoring system may aid in the retrospective identification of thrombophilia-related placental pathology and support the integration of genetic and histologic data in perinatal risk assessment.

## 1. Introduction

Thrombophilia, a predisposition to thrombosis due to inherited or acquired coagulation abnormalities, has been increasingly recognized as a contributor to adverse pregnancy outcomes [1,2,3]. Inherited thrombophilic mutations such as Factor V Leiden and Prothrombin G20210A, along with other commonly investigated genetic polymorphisms of uncertain clinical significance (e.g., MTHFR variants, PAI-1 4G/4G, Factor XIII V34L), have been associated in the literature with placental vascular dysfunction, which may contribute to fetal growth restriction, preeclampsia, placental abruption, and stillbirth [4,5]. These mutations are known to affect maternal–fetal circulation by promoting microthrombi formation and altering vascular remodeling during placentation [6,7].

Although previous studies have explored associations between thrombophilia and clinical outcomes, fewer have systematically examined the histopathological characteristics of the placenta in genetically stratified cohorts [2,8]. Placental lesions such as infarctions, intervillous thrombosis, acute atherosis, and villous stasis reflect underlying hemodynamic disturbances and can serve as morphological markers of thrombophilic influence on pregnancy [9,10]. The umbilical cord is vital to fetal development, yet specific features like cord coiling remain underexplored. Coiling, quantified by the umbilical coiling index, is a physiological adaptation that helps protect the cord from external pressure [11]. In parallel, the structure of the umbilical cord, including coiling pattern, insertion site, and hypertwisting, may also provide insight into fetal adaptive mechanisms in response to vascular stress [11,12,13,14].

This study aimed to evaluate the relationship between inherited thrombophilic mutations and histological features of the placenta and umbilical cord. By comparing placental pathology and morphometric parameters in women with and without thrombophilia, we sought to clarify the role of genetic prothrombotic risk in placental vascular compromise and fetal development. The findings may contribute to improved risk stratification, targeted screening, and early intervention strategies in thrombophilia-associated pregnancies.

It is important to acknowledge that certain polymorphisms included in this study—such as *MTHFR C677T*, *PAI-1 4G/4G*, and *Factor XIII V34L*—have been the subject of substantial debate within the scientific community. While these variants have historically been associated with adverse pregnancy outcomes, recent expert consensus has questioned their clinical relevance in the context of thrombophilia screening. Notably, current guidelines explicitly discourage the routine testing of MTHFR polymorphisms, citing insufficient evidence of a causal link with thrombotic events or pregnancy complications. In light of these developments, the present study does not seek to endorse the clinical utility of these markers. Instead, their inclusion serves a purely exploratory purpose—to investigate potential associations between these common genetic variants and subclinical placental histopathological changes in a genetically stratified cohort [15].

## 2. Materials and Methods

### 2.1. Study Design and Ethical Approval

**Figure 1:**
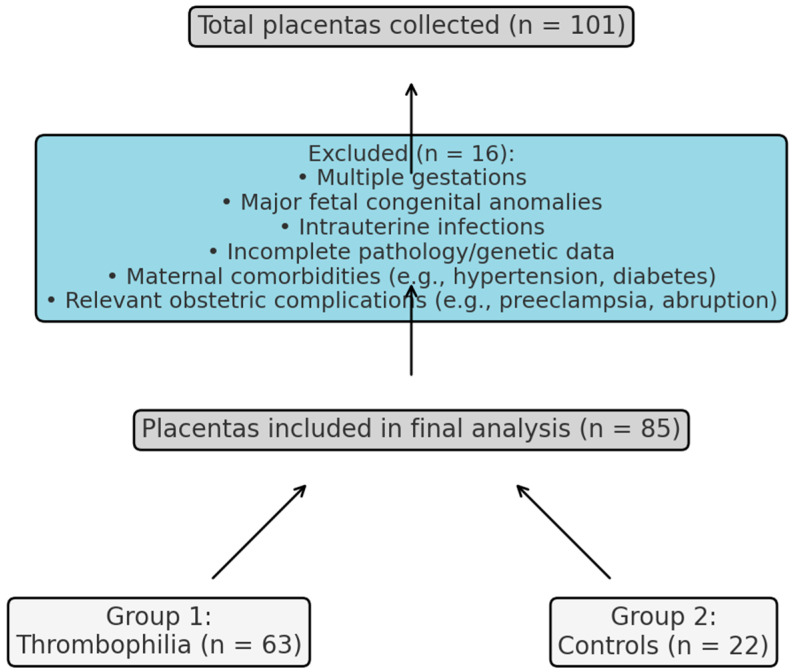
Sample flow diagram showing initial collection, exclusion criteria, and final group allocation.

This retrospective observational study included 101 placental specimens collected at the Clinical County Emergency Hospital Bihor, Romania, between September 2020 and September 2024. After applying exclusion criteria, 85 placentas were eligible for analysis: 63 assigned to the thrombophilia group (Group 1), based on confirmed inherited prothrombotic mutations, and 22 to the control group (Group 2), from pregnancies without known maternal pathology. No formal a priori power calculation was performed; however, a post hoc estimation based on our final sample (*N* = 85; 63 thrombophilia, 22 controls) suggests approximately 80% power to detect a moderate-to-large effect size (Cohen’s d ≈ 0.7) at α = 0.05. The selection process and final allocation of placentas are detailed in Figure 1. The study protocol was approved by the local Institutional Review Board (IRB), and all procedures complied with the Declaration of Helsinki. Written informed consent was obtained from all participants before inclusion.

### 2.2. Study Population

Inclusion criteria for both groups encompassed the availability of complete placental inclusion criteria for both groups included the availability of complete placental assessment (macroscopic and histologic), validated genetic testing, and comprehensive clinical records with antenatal follow-up.

Exclusion criteria encompassed multiple gestations, major fetal congenital anomalies, intrauterine infections, incomplete pathology or genetic data, and known confounding maternal conditions that could influence placental morphology or vascular pathology. These included the following:

- Systemic comorbidities (e.g., chronic hypertension, diabetes mellitus, autoimmune diseases);
- Relevant obstetric complications (e.g., preeclampsia, placenta previa, placental abruption, preterm premature rupture of membranes).

Group 1 comprised placentas from women with confirmed inherited thrombophilic mutations, identified through standardized molecular testing. Group 2 included placentas from term or late preterm pregnancies without maternal pathology, genetic thrombophilia, or pregnancy complications.

### 2.3. Placental Collection and Macroscopic Examination

Placental samples were collected from all eligible term deliveries between January 2022 and December 2023, with 101 cases screened in total before applying exclusion criteria. Placentas were collected immediately after delivery and fixed in 10% neutral buffered formalin. A standardized protocol was applied for macroscopic assessment by a dedicated pathology team blinded to maternal thrombophilia status. Measured parameters included the following:

Placental weight (grams);
Maximum placental diameter (centimeters);
Placental thickness (centimeters);
Umbilical cord length (centimeters) and insertion type (central, eccentric, or marginal).

### 2.4. Histopathological Evaluation

Histological assessments were performed within the Department of Pathology at the Bihor County Emergency Clinical Hospital by pathologists experienced in placental evaluation. The observers were blinded to thrombophilia status, clinical outcomes, and study group allocation. Only maternal age and gestational age, routinely included in pathology forms, were available to guide lesion interpretation.

Sections representative of central and peripheral cotyledons, the umbilical cord, and membranes were processed and stained with hematoxylin and eosin. Histologic features were scored according to the Amsterdam Consensus (2016), classifying lesions under Maternal Vascular Malperfusion (MVM) or Fetal Vascular Malperfusion (FVM) where appropriate. Key lesions recorded included:

• Intervillous thrombi (excessive: MVM);
• Placental infarction (MVM);
• Decidual vasculopathy (acute atherosis; MVM);
• Villous stasis/delayed villous maturation (MVM);
• Stromal fibrosis (mild nonspecific; Other/MVM);
• Avascular or thrombosed fetal vessels (FVM).

A detailed overview of the placental lesions assessed, their classification under the Amsterdam Consensus criteria, and inclusion in the composite histological score is presented in Table 1.

Morphometric analysis was performed on images acquired at 40× magnification using a standard light microscope. Vascular profiles within terminal villi were manually selected and measured by a single observer using ImageJ (Version 1.54p, NIH, USA), following a predefined protocol. Vessel inclusion criteria included regular shape, intact endothelial lining, and localization within terminal villi. To ensure consistency, measurements were repeated in a random subset of images, with acceptable intra-observer variability. A schematic of the analysis workflow and a representative example are provided in Supplementary Figure S1.

### 2.5. Histologic Score Development

While established scoring systems for maternal vascular malperfusion—such as the Biron Shental index and the Khong MVM severity grading system—provide comprehensive assessment frameworks, they often rely on morphometric or semiquantitative parameters that are not consistently feasible in retrospective cohorts. To ensure applicability in routine histopathological evaluation, we developed a streamlined five-item composite score, aligned with the Amsterdam Consensus criteria.

The five lesions included—villous stasis, stromal fibrosis, intervillous thrombosis, placental infarction, and acute atherosis—were selected based on their frequent documentation in thrombophilia-associated placentas, as well as their reproducibility and pathophysiological relevance in maternal vascular malperfusion. These features have been repeatedly linked in the literature to altered uteroplacental circulation in thrombophilic pregnancies and were consistently observed in our cohort.

**Figure 2:**
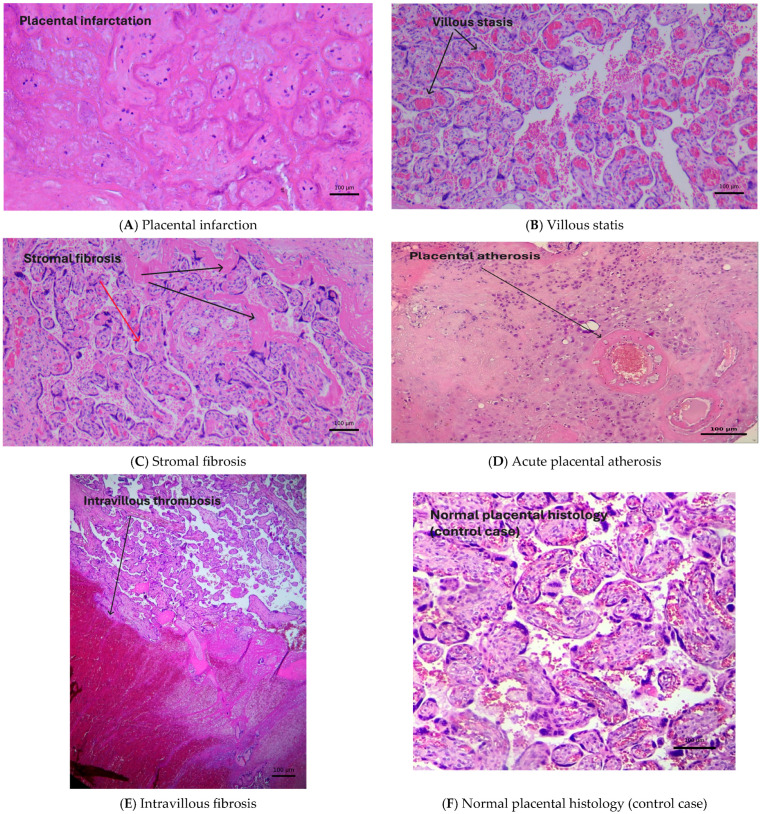
Histopathologic features showing (**A**) placental infarction, (**B**) villous stasis, (**C**) stromal fibrosis, (**D**) acute placental atherosis, (**E**) intravillous fibrosis, and (**F**) normal placental histology (control case).

One point was assigned for the presence of each lesion, resulting in a total score ranging from 0 to 5. A cutoff value of ≥3 was pre-defined and explored for its discriminatory capacity between the thrombophilia and control groups. Internal validation showed a statistically significant association between scores ≥3 and known thrombophilic mutations. Although interobserver agreement was not formally tested, all assessments were performed within the Department of Pathology by experienced placental pathologists using standardized diagnostic criteria. The histopathologic findings are presented in Figure 2.

### 2.6. Genetic Evaluation

Genetic testing had been performed before pregnancy in all women included in the thrombophilia group, based on clinical indications or family history. Genetic results were retrieved from patients’ medical records and confirmed to follow standardized diagnostic protocols. Peripheral blood samples had been collected at the time of initial evaluation, and DNA extraction was performed using silica-based column kits (QIAamp DNA Blood Mini Kit, Qiagen, Hilden, Germany; Cat. No. 51104) or automated extraction platforms. Genotyping was conducted by real-time polymerase chain reaction (PCR) using allele-specific primers and fluorescent probes (TaqMan chemistry, Coralville, IA, USA). Internal positive and negative controls were included in each run to ensure validity. The panel included the following: Factor V Leiden (G1691A), Prothrombin gene mutation (G20210A), MTHFR C677T, MTHFR A1298C, PAI-1 4G/5G polymorphism, Factor XIII V34L, and EPCR a1/a3 alleles. All analyses were conducted in an ISO-certified molecular diagnostics laboratory, and results were categorized as homozygous, heterozygous, or wild-type for each locus. Genetic data were then correlated with histopathological findings and clinical outcomes.

### 2.7. Statistical Analysis

Analyses were performed using SPSS (v20, Chicago, IL, USA) with SciPy and statsmodels libraries. Continuous variables were tested for normality using the Shapiro–Wilk test and compared by Student’s *t*-test or Mann–Whitney U test as appropriate. Categorical data were analyzed with a Chi-square test or Fisher’s exact test. Associations between the histologic score and continuous clinical or macroscopic parameters were evaluated with Pearson or Spearman correlation coefficients. A multivariate linear regression model identified independent predictors of the histologic score. Statistical significance was set at *p* < 0.05.

## 3. Results

### 3.1. Demographic and Birth Data—Summary

**Figure 3:**
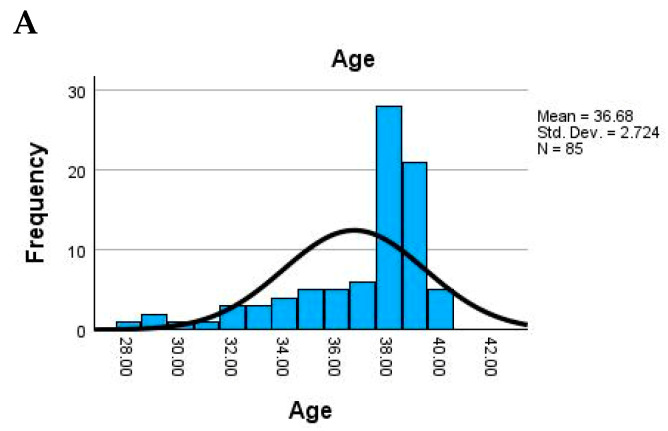

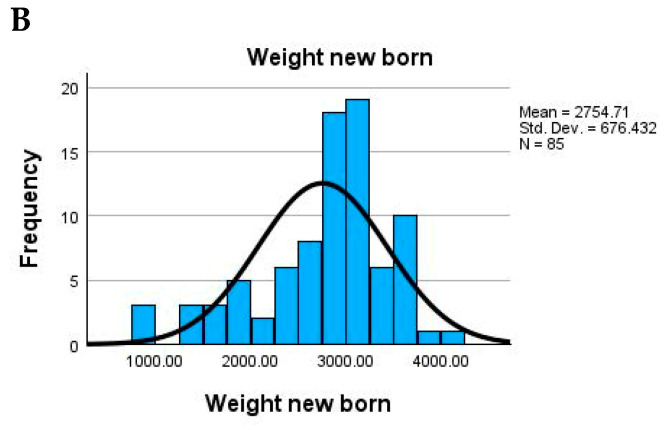

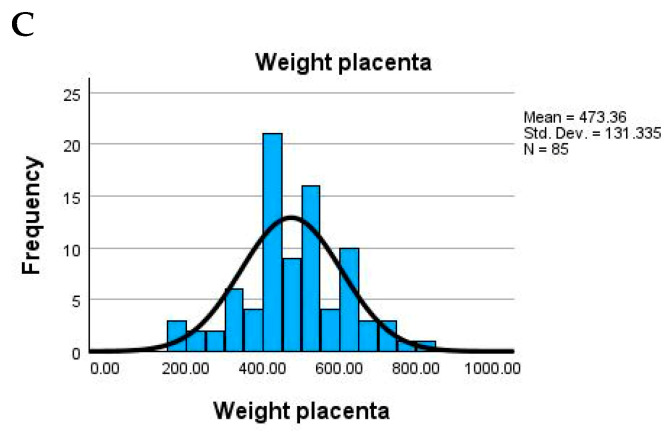

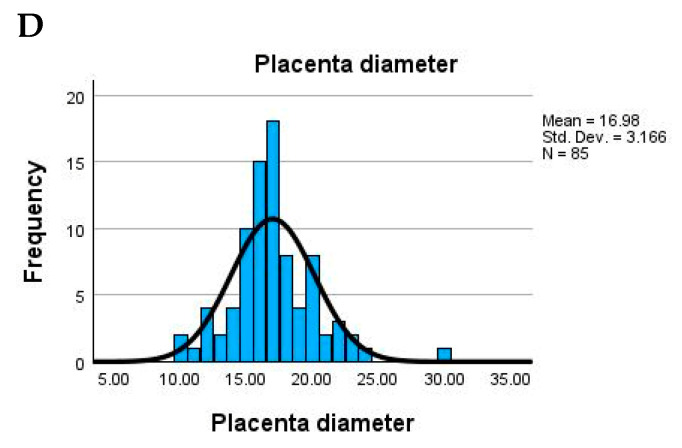
Distribution of general maternal and placental parameters. (**A**) Histogram of maternal age, showing a slightly left-skewed distribution with a peak around 37 years. (**B**) Histogram of newborn weight, demonstrating a broad distribution from low to normal birth weight (mean ≈ 2755 g). (**C**) Histogram of placental weight, revealing a near-normal distribution centered around 473 g. (**D**) Histogram of placental diameter, showing a narrow peak around 17 cm with a few outliers. All distributions are accompanied by a kernel density estimate curve and report mean, standard deviation, and sample size (*N*= 85).

The study population consisted of adult women with a relatively narrow age range, the mean maternal age being 36.7 years (SD = 2.7), suggesting a homogeneous group. Newborn weight showed substantial variability, with an average of 2754.7 g and values ranging from very low birth weight (900 g) to normal (up to 4000 g). The placental weight also varied widely (mean = 473.4 g), possibly indicating underlying pathological changes in some cases (Figure 3).

Placental size parameters were generally within expected limits. The average placental diameter was 17 cm (range: 10–30 cm), while thickness averaged 2.3 cm, with some extreme values (as low as 0 cm), potentially pointing to measurement issues or anomalies. The umbilical cord also exhibited significant variation: average length was 19.6 cm (ranging from 0 to 45 cm), and width averaged 1.13 cm. A minimum width or length of 0 could suggest missing data or abnormal development. The descriptive statistics of maternal and birth-related parameters in the study population, and a complete mapping of original descriptive terms to the Amsterdam classification is presented in Table 1.

### *3.2*. *Baseline Characteristics*

The baseline characteristics of the study participants are summarized in Table 2. No statistically significant differences were observed between the thrombophilia and control groups regarding gestational age at delivery (36.5 ± 2.6 vs. 36.9 ± 3.1 weeks, *p* = 0.2042), fetal birth weight (2754.7 ± 698.2 g vs. 2770.9 ± 630.4 g, *p* = 0.8346), or placental macroscopic parameters. Specifically, mean placental weight, diameter, and thickness were comparable between groups, with *p*-values > 0.4 in all comparisons. These findings suggest that gross placental morphology may not reliably differentiate pregnancies affected by thrombophilia from those without thrombotic risk, thereby highlighting the potential role of histopathological examination in detecting subclinical placental alterations.

Effect size analysis using Cohen’s d (Table 2) revealed large differences between thrombophilia and control groups for several genetic mutations, including PAI-1 4G/4G (*d* = 1.91), MTHFR C677T (*d* = 1.85), EPCR (*d* = 1.06), and Factor V Leiden (*d* = 0.93), suggesting strong associations with thrombophilic status. Moderate to large effect sizes were also observed for placental lesions such as villous stasis (*d* = 0.65), acute atherosis (*d* = 0.60), and retroplacental hematoma (*d* = 0.68), supporting the hypothesis that histopathological features reflect underlying thrombotic risk. Behavioral factors like smoking also showed a moderate effect (*d* = 0.55), indicating potential lifestyle contributions to placental vascular dysfunction. These findings emphasize the potential utility of combining histological and genetic markers in evaluating thrombophilic risk.

The cohort was divided into two groups: individuals with at least one genetic mutation (Group 1) and those without mutations (Group 2). Overall, there were no statistically significant differences between the groups across the assessed parameters. Maternal age, newborn weight, placental weight and dimensions, as well as umbilical cord length and width, were all comparable between the two groups (*p* > 0.05 for all). These findings suggest that the presence of genetic mutations did not significantly influence basic maternal or neonatal anthropometric characteristics in this sample. The comparison of maternal and placental morphometric parameters between thrombophilia and control groups is presented in Table 3.

### 3.3. Genetic Polymorphisms (Heterozygote/Homozygote Values Range from 0 to 2)

The study assessed a panel of genetic variants, including both clinically validated thrombophilia markers (Factor V Leiden and Prothrombin G20210A) and additional polymorphisms frequently investigated in relation to pregnancy outcomes, such as MTHFR C677T, MTHFR A1298C, PAI-1 4G/4G, Factor XIII V34L, and EPCR variants. The most prevalent polymorphism in the cohort was PAI-1 4G/4G (mean = 0.98), followed by MTHFR C677T (mean = 0.76) and MTHFR A1298C (0.48). Factor V Leiden and Factor XIII V34L were present in approximately 26–48% of individuals, while Factor II G20210A and EPCR variants were less frequent (means ~0.26 and 0.34).

Placental pathology findings showed several common lesions. Intervillous thrombosis, villous agglutinations, and staza vilozitară (villous stasis) were each observed in around 38–50% of cases, suggesting widespread circulatory disturbances. Placental infarctions, acute atherosis, and marked villous edema were also common, each appearing in roughly a third of the sample. Inflammatory and ischemic findings such as chorioamnionitis, acute hypoxia/malperfusion, and retroplacental hematoma were present in 18–26% of placentas. Features like immature or fibrous villous appearance and hypertwisting of the cord were variably expressed, pointing to diverse placental responses to maternal and fetal stress.

A statistically significant difference was observed in the distribution of all analyzed genetic mutations between the two groups (*p* < 0.05 for all variants). In Group 1 (with mutations), heterozygous and homozygous forms of Factor V Leiden, Factor II G20210A, MTHFR C677T, MTHFR A1298C, Factor XIII V34L, PAI-1 4G/4G, and EPCR variants were present in varying proportions. In contrast, Group 2 (without mutations) included only individuals with no detectable mutation for any of the investigated thrombophilia markers. These findings confirm the clear genetic separation between the two groups and validate the classification used in the study (Table 4).

**Figure 4:**
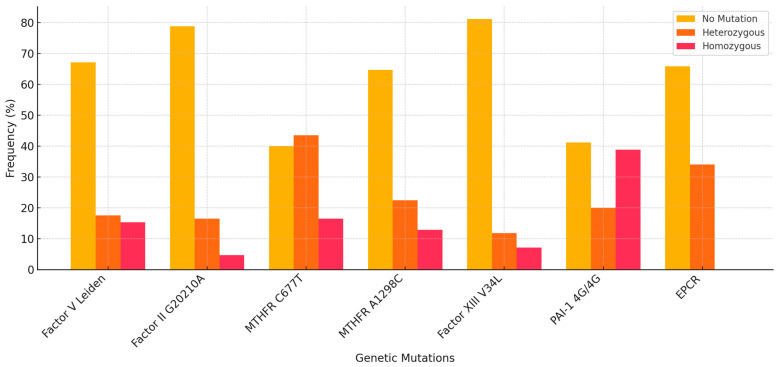
Distribution of thrombophilic genetic mutations.

The distribution of thrombophilic genetic mutations is presented in Figure 4.

### 3.4. Placental and Cord Pathology Features

Histopathological examination of the placentas revealed several frequent abnormalities. Staza vilozitară (villous stasis) and villous agglutinations were the most common, present in approximately 50% and 45% of cases, respectively, indicating significant circulatory stagnation and clustering of villi. Intervillous thrombosis and placental infarction were also prevalent (each around 38%), suggesting widespread disturbances in maternal-fetal blood flow. Marked villous edema (39%) and acute hypoxia or malperfusion (35%) pointed to signs of fetal vascular compromise.

Chronic lesions such as fibrous appearance (34%) and acute placental atherosis (34%) indicated long-term vascular stress or damage, possibly associated with maternal conditions like hypertension, which was noted in 22% of cases. Inflammatory changes were observed less frequently, with chorioamniotitis present in 18% of samples. Other findings included retroplacental hematoma (26%), boiled meat appearance (27%)—a nonspecific macroscopic marker of pathology—and immature villous structure (16%), which may suggest delayed placental development. Chorionic vasal thrombosis, while less common (13%), represents a severe vascular lesion when present (Table 5).

**Table 5:** Prevalence and interpretation of thrombophilia-related genetic mutations in the study cohort.

Variable	Mean	Interpretation
Factor V Leiden	0.48	Approx. 48% of the sample carries at least one mutated allele.
Factor II G20210A	0.26	About 26% have mutation, suggesting a moderate prevalence.
MTHFR C677T	0.76	Over 75% show some variant.
MTHFR A1298C	0.48	Roughly half have a mutation.
Factor XIII V34L	0.26	Mutation present in about 26%.
PAI-1 4G/4G	0.98	Very high frequency—nearly all subjects carry this variant.
EPCR (endothelial protein C receptor)	0.34	Detected in about 1/3 of the cohort.

When comparing placental pathology between the two groups, several lesions were more frequent in individuals with genetic mutations (Group 1), although not all reached statistical significance. Notably, acute placental atherosis, retroplacental hematoma, signs of maternal hypertension, and villous stasis were significantly more common in Group 1 (*p* < 0.05), suggesting a link between thrombophilic mutations and maternal vascular malperfusion. Other findings, such as boiled meat appearance, fibrous changes, placental infarction, and villous edema, were also more frequent in Group 1 but did not reach statistical significance. Inflammatory changes like chorioamnionitis and features such as chorionic vasal thrombosis and intervillous thrombosis showed no meaningful differences between groups. Overall, the presence of thrombophilia appears to be associated with a higher rate of placental vascular lesions (Table 6).

### 3.5. Umbilical Cord Features

Assessment of umbilical cord characteristics revealed that non-central insertion was common, with a mean value of 0.72, indicating that most cords were marginal or eccentrically inserted. Approximately 39% of cases showed hypertwisting of the cord, a feature that may impact fetal blood flow or indicate intrauterine stress. Additionally, normal cord coiling was present in 72% of cases, although some variation was observed, suggesting mild structural abnormalities in a subset of the cohort (Table 7).

When analyzing umbilical cord characteristics, insertion type (central, eccentric, or marginal) did not differ significantly between groups (*p* = 0.281), with central insertion being most common overall (45%). However, significant differences were observed for both cord twisting and coiling. Hypertwisting was more frequent in the group without genetic mutations (Group 2), present in 17.6% compared to 21.2% in Group 1 (*p* = 0.001), suggesting a possible compensatory or unrelated mechanical factor. Similarly, modified (abnormal) cord coiling was significantly more common in Group 1 (60%) versus 11.8% in Group 2 (*p* = 0.001), indicating a potential association between thrombophilia and altered cord morphology (Table 8).

**Figure 5:**
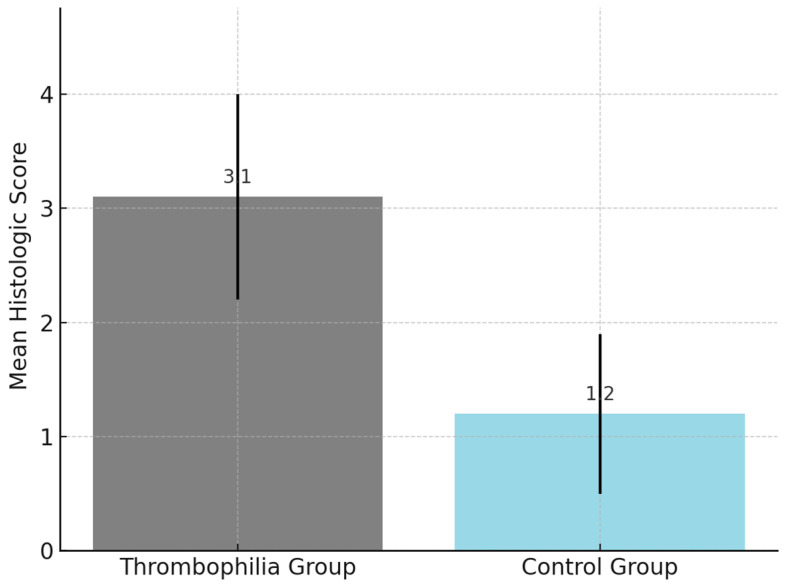
Comparison of composite histologic scores between groups.

The comparison of composite histologic scores between groups is presented in Figure 5.

### 3.6. Correlations with General Aspects

Correlation analysis between genetic mutations and general parameters revealed no significant associations with maternal age. However, Factor II G20210A showed weak but statistically significant negative correlations with newborn weight (*r* = −0.231, *p* = 0.034) and placental weight (*r* = −0.249, *p* = 0.022), suggesting a possible impact on fetal growth. Similarly, Factor V and Factor II were both negatively correlated with placental diameter, and Factor V also with placental thickness, indicating that these mutations may be linked to subtle placental structural changes. Other correlations were weak and not statistically significant (Table 9).

### 3.7. Correlations with Genetic Mutations

Several significant correlations were observed between the studied genetic mutations. Factor V was strongly correlated with Factor II G20210A (*r* = 0.720, *p* < 0.001), suggesting they frequently co-occur. Factor V also showed moderate associations with Factor XIII V34L and PAI-1 4G/4G. MTHFR C677T was significantly correlated with both PAI-1 4G/4G and EPCR, indicating a possible shared genetic predisposition. Similarly, MTHFR A1298C correlated with PAI-1 4G/4G and EPCR, while PAI-1 4G/4G itself showed multiple associations, including with Factor XIII V34L. These patterns suggest clusters of co-inherited thrombophilic mutations in the cohort (Table 10).

### 3.8. Correlations with Placenta Appearance

Several genetic mutations were significantly associated with placental morphological changes. Factor V showed strong positive correlations with placental infarction, fibrous appearance, villous agglutinations, and intervillous thrombosis, suggesting a vascular impact. Factor II G20210A was also positively correlated with placental infarction, intervillous thrombosis, and chorionic vasal thrombosis. MTHFR C677T was weakly associated with villous edema, signs of maternal hypertension, and villous stasis, while MTHFR A1298C showed a mild link to boiled meat appearance. Factor XIII V34L had the strongest associations, significantly correlating with placental infarction, atherosis, and hypertension. PAI-1 4G/4G was linked to signs of hypertension, villous stasis, and villous edema, whereas EPCR showed an inverse correlation with placental infarction and intervillous thrombosis, but a positive one with villous edema and villous stasis. These findings suggest distinct vascular and inflammatory placental patterns associated with specific thrombophilic mutations (Table 11).

### 3.9. Correlations with the Umbilical Cord

Most genetic mutations showed no significant correlation with umbilical cord length, width, or insertion site, indicating that these features are largely independent of thrombophilic status. However, he following notable associations emerged: MTHFR C677T was negatively correlated with hypertwisted cords (*r* = −0.245, *p* = 0.024), suggesting reduced twisting in carriers. PAI-1 4G/4G showed a strong negative correlation with hypertwisting (*r* = −0.303, *p* = 0.005), and a positive correlation with normal cord coiling (*r* = 0.363, *p* = 0.001), implying a protective or stabilizing effect. Additionally, MTHFR A1298C was positively correlated with normal coiling (*r* = 0.278, *p* = 0.010). These findings suggest certain genetic variants may subtly influence cord structure, particularly in relation to coiling and twisting (Table 12).

The correlations between thrombophilia-associated genetic mutations and placental histopathological lesions are presented in Table 13.

**Table 13:** Correlations between thrombophilia-associated genetic mutations and placental histopathological lesions.

Parameters		Factor_V	Factor II G20220A	MTHFR C677T	MTHFR A1298C	Factor XIII V34L	PAI 1 4G4G	EPCR
Boiled meat	*r*	0.210	0.101	0.052	0.219 *	0.094	0.046	0.176
	*p*	0.054	0.356	0.634	0.044	0.392	0.678	0.107
Immature appearance	*r*	0.011	0.082	−0.120	0.100	−0.034	0.154	−0.119
	*p*	0.924	0.458	0.273	0.363	0.756	0.161	0.279
Fibrous appearance	*r*	0.267 *	0.116	0.202	0.070	0.150	0.130	−0.047
	*p*	0.014	0.292	0.063	0.524	0.170	0.236	0.671
Placental infarction	*r*	0.442 **	0.350 **	0.120	0.087	0.535 **	0.211	−0.354 **
	*p*	0.000	0.001	0.274	0.427	0.000	0.053	0.001
Acute placental atherosis	*r*	0.200	0.209	0.168	−0.104	0.279 **	0.213	−0.099
	*p*	0.066	0.055	0.125	0.344	0.010	0.050	0.367
Marked villous edema	*r*	0.067	0.111	0.229 *	0.104	0.019	0.048	0.292 **
	*p*	0.540	0.312	0.035	0.342	0.862	0.663	0.007
Signs of HTN	*r*	0.183	0.163	0.256 *	−0.086	0.298 **	0.267 *	−0.029
	*p*	0.093	0.137	0.018	0.435	0.006	0.014	0.794
Acute hypoxia malperfusion	*r*	−0.049	−0.081	0.209	0.018	0.138	0.130	−0.012
	*p*	0.659	0.460	0.055	0.868	0.208	0.237	0.912
Chorioamniotitis	*r*	0.032	0.007	−0.193	−0.010	0.060	−0.091	0.057
	*p*	0.774	0.951	0.076	0.926	0.587	0.406	0.602
Retroplacental hematoma	*r*	−0.094	−0.035	0.195	0.015	0.014	0.106	−0.029
	*p*	0.392	0.751	0.074	0.894	0.897	0.336	0.795
Intervillous thrombosis	*r*	0.540 **	0.487 **	−0.118	0.087	0.241 *	0.129	−0.303 **
	*p*	0.000	0.000	0.282	0.427	0.027	0.239	0.005
Chorionic vassal thrombosis	*r*	0.362 **	0.272 *	−0.069	−0.064	0.131	−0.107	−0.056
	*p*	0.001	0.012	0.528	0.559	0.233	0.328	0.613
Villous agglutinations	*r*	0.275 *	0.229 *	0.164	0.055	0.130	0.077	0.102
	*p*	0.011	0.035	0.134	0.614	0.237	0.486	0.355
Villous stasis	*r*	0.071	0.126	0.268 *	0.207	0.199	0.263 *	0.265 *
	*p*	0.517	0.249	0.013	0.058	0.068	0.015	0.014
	*N*	85	85	85	85	85	85	85

*r* = Pearson coefficient, *p* = statistical significance, *N* = number of patients, ** = correlation is significant at the 0.01 level (2-tailed), * = correlation is significant at the 0.05 level (2-tailed).

The correlations between thrombophilic genetic mutations and umbilical cord morphological features are presented in Table 14.

**Table 14:** Correlations between thrombophilic genetic mutations and umbilical cord morphological features.

Parameters		Factor V	Factor II G20220A	MTHFR C677T	MTHFR A1298C	FACTOR XIII V34L	PAI/1 4G4G	EPCR
Umbilical cord length	*r*	−0.055	−0.019	−0.052	0.031	0.105	−0.143	−0.014
	*p*	0.619	0.861	0.634	0.779	0.340	0.190	0.899
Umbilical cord width	*r*	−0.093	−0.181	0.090	−0.064	0.118	0.025	−0.106
	*p*	0.398	0.098	0.412	0.561	0.281	0.821	0.336
Inset	*r*	−0.052	−0.114	−0.037	0.081	−0.134	−0.136	−0.062
	*p*	0.634	0.298	0.735	0.462	0.222	0.213	0.575
Hypertwisted	*r*	−0.094	−0.024	−0.245 *	−0.166	−0.106	−0.303 **	−0.013
	*p*	0.390	0.824	0.024	0.128	0.333	0.005	0.905
CO normal	*r*	0.090	0.108	0.123	0.278 *	0.010	0.363 **	0.066
	*p*	0.411	0.325	0.263	0.010	0.931	0.001	0.551
	*N*	85	85	85	85	85	85	85

*r* = Pearson coefficient, *p* = statistical significance, *N* = number of patients, ** = correlation is significant at the 0.01 level (2-tailed), * = correlation is significant at the 0.05 level (2-tailed).

### 3.10. Multiple Linear Regression

A multiple linear regression adjusting for smoking, BMI, gestational age, and parity showed that thrombophilia was independently associated with higher histologic scores (β = +2.3; 95% CI = 1.1–3.5; *p* = 0.001). Gestational age was inversely associated with histologic score (β = –0.2; 95% CI = –0.4–0.0; *p* = 0.045). Other covariates were not significant predictors (Table 15).

These results confirm that thrombophilia status is independently associated with higher placental pathology scores, even after adjusting for smoking, BMI, gestational age, and parity.

**Figure 6:**
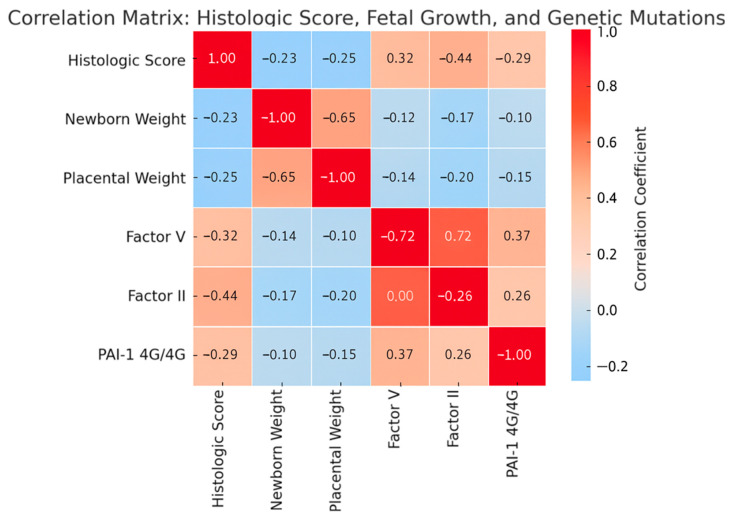
Correlation matrix: histologic score, fetal growth, and genetic mutations.

In addition to the regression analysis, the correlation matrix (Figure 6) further supports these associations. Positive correlations were observed between the composite histologic score and thrombophilic genetic mutations (Factor V Leiden, Factor II G20210A, and PAI-1 4G/4G), suggesting a link between these variants and increased severity of placental vascular lesions. Conversely, negative correlations with newborn and placental weight highlight the impact of histological damage on fetal growth. These findings underscore the interplay between thrombophilia status, placental pathology, and fetal outcomes, complementing the adjusted regression results.

## 4. Discussion

This study investigated the relationship between inherited thrombophilic mutations and placental structural abnormalities, aiming to understand the histopathological footprint of thrombophilia in pregnancies without overt clinical complications. The findings demonstrate that placentas from women with hereditary thrombophilia exhibit a significantly higher frequency of vascular lesions, including infarctions, acute atherosis, intervillous thrombosis, stromal fibrosis, and villous stasis—hallmarks of maternal vascular malperfusion (MVM) [16,17,18,19,20]. These results are consistent with and extend existing literature on thrombophilia-related placental dysfunction.

Previous studies have reported similar associations between Factor V Leiden or Prothrombin G20210A mutations and increased rates of placental infarction and decidual vasculopathy [21,22,23,24]. Our results confirm this link and further demonstrate that these mutations correlate with measurable changes in placental morphology, including reduced placental diameter and thickness, even in the absence of clinical complications such as preeclampsia or intrauterine growth restriction (IUGR). In line with the work by Gris et al. [25], we observed that even asymptomatic thrombophilic women may exhibit subclinical placental damage, supporting the idea that genetic predisposition alone can impair placental perfusion [26,27,28].

These findings underscore the need to explore the pathophysiological mechanisms by which inherited prothrombotic mutations affect placental vascular development. The proposed model suggests that mutations such as *Factor V Leiden* and *Prothrombin G20210A* induce a hypercoagulable maternal state, favoring the formation of microthrombi within the intervillous and fetal vascular compartments. These thrombotic events can compromise oxygen and nutrient exchange, leading to regional hypoxia, impaired spiral artery remodeling, and altered angiogenesis. As a result, the placenta may develop chronic vascular lesions such as infarctions, villous hypovascularity, and acute atherosis—even in clinically uncomplicated pregnancies. These histopathological features reflect subclinical maternal vascular malperfusion and may represent an early or compensated phase of placental insufficiency.

The high prevalence of PAI-1 4G/4G and MTHFR polymorphisms in our cohort and their association with villous edema, delayed maturation, and abnormal cord coiling patterns aligns with findings by Kupferminc et al. [29] and Pasta et al. [30], who noted similar morphological disturbances in placentas from thrombophilic pregnancies [31,32,33,34,35]. Notably, our study is among the few to report significant correlations between these mutations and umbilical cord features—such as abnormal coiling and hypertwisting—which may represent subtle indicators of fetal adaptation to vascular stress. Nonetheless, these associations warrant cautious interpretation, as contemporary evidence does not substantiate the clinical utility of *MTHFR* and *PAI-1* genotyping within standard thrombophilia screening protocols.

It is important to acknowledge that the high prevalence of certain mutations in our thrombophilia group—particularly PAI-1 4G/4G (98%) and MTHFR C677T (76%)—substantially exceeds reference frequencies reported in the general European population. This discrepancy likely reflects a selection bias, as participants in this group underwent genetic testing based on clinical suspicion or obstetric history rather than as part of routine population screening. Consequently, the reported frequencies should not be interpreted as representative of the Romanian population at large. Future studies incorporating matched population-level controls are warranted to determine the true distribution and clinical significance of these variants in our region.

Furthermore, the use of a composite histologic score allowed for the semi-quantitative assessment of thrombophilia-associated placental damage. A cutoff value of ≥3 effectively distinguished between the thrombophilia and control groups, suggesting its potential as a histological screening tool, particularly in cases lacking clinical suspicion.

The composite histologic score developed in this study, while designed to reflect reproducible and clinically relevant lesions, has not yet been validated in external populations. Future prospective studies are needed to assess its predictive performance and applicability across diverse clinical settings.

Our findings suggest that the proposed composite histological score, based on reproducible features of maternal vascular malperfusion, may aid in identifying placental patterns commonly associated with inherited thrombophilia. Incorporating such assessments into routine postnatal placental evaluation could improve risk stratification and support more personalized care in subsequent pregnancies.

Importantly, this score may also serve as a prospective trigger for targeted genetic testing. In cases where women experience serious obstetric complications—such as preeclampsia, fetal growth restriction, or placental abruption—without a known thrombophilia diagnosis, a high placental score may indicate an underlying prothrombotic condition. In such settings, histopathological findings could guide postnatal genetic screenings, ensuring earlier identification of thrombophilic mutations and enabling preventive strategies for future pregnancies or other hypercoagulable conditions.

While the primary focus of this study was on placental morphology in the context of maternal thrombophilia, available neonatal data were retrospectively reviewed. Most newborns had favorable short-term outcomes, and no consistent pattern of acute or chronic morbidity could be established based on genotype or histological findings. Due to limited long-term follow-up and the multifactorial nature of neonatal outcomes, a direct association between specific thrombophilic mutations or placental lesions and neonatal complications could not be reliably determined. Further prospective studies are required to assess the clinical impact of these findings on offspring health.

We acknowledge that smoking prevalence was significantly higher in the thrombophilia group (33% vs. 5%), representing a potential confounding factor for certain placental lesions. While our sample size limited formal multivariable adjustment, sensitivity analyses excluding smokers showed broadly consistent patterns of histopathologic differences, although residual confounding cannot be fully excluded. This limitation underscores the need for larger studies to disentangle the independent effects of thrombophilia and smoking on placental pathology.

### Limitations

This study is subject to several limitations that warrant consideration. First, its retrospective design inherently limits the ability to establish causal relationships between inherited thrombophilic mutations and placental abnormalities. Second, the relatively modest sample size may affect both the generalizability of findings and the statistical power to detect more nuanced associations. Third, despite the blinding of pathologists to group allocation, the qualitative nature of certain histopathological assessments introduces the possibility of observer bias. Moreover, the study did not control for potential confounding variables such as maternal treatments—particularly anticoagulant therapy or folate supplementation—that may influence placental morphology.

An additional limitation lies in the scope of the genetic analysis, which was confined to a predefined panel of mutations, thereby excluding rarer or recently identified variants that may also play a role in placental pathology. Of particular note is the inclusion of certain polymorphisms, such as *MTHFR* C677T and *PAI-1* 4G/4G, which are no longer regarded as clinically relevant markers of inherited thrombophilia under current international guidelines. While these variants were historically investigated in the context of pregnancy complications, their thrombotic significance is not supported by contemporary evidence, and their use in clinical screening is presently discouraged. Their presence in the current analysis reflects both the retrospective character of the cohort and the historical prevalence of these tests in local diagnostic protocols. Importantly, the study does not endorse their clinical utility but rather incorporates them to provide a broader exploratory perspective on the potential relationships between commonly encountered genetic polymorphisms and placental histopathology.

## 5. Conclusions

This study provides evidence of a significant association between inherited prothrombotic genetic variants and histopathological markers of placental vascular compromise. Mutations such as Factor V Leiden and Prothrombin G20210A—recognized contributors to thrombotic risk—were found to correlate with a higher prevalence of placental infarctions, acute atherosis, intervillous thrombosis, and villous stasis, consistent with features of maternal vascular malperfusion. Notably, comparable clinical parameters between groups, including neonatal weight and placental dimensions, suggest that such histological alterations may precede or occur independently of overt fetal growth impairment.

Furthermore, exploratory associations between polymorphisms such as *PAI-1 4G/4G* and *MTHFR A1298C* and structural features of the umbilical cord—specifically coiling anomalies—may reflect subtle developmental responses to altered intrauterine hemodynamics. These findings, however, should be interpreted with caution. Several of the investigated variants, particularly *MTHFR C677T* and *PAI-1 4G/4G*, are no longer endorsed as clinically actionable thrombophilia markers in current consensus guidelines. Their inclusion in this study is intended to expand the understanding of potential morphopathological correlations rather than to imply diagnostic or therapeutic value.

In conclusion, while our results highlight histological patterns potentially linked to genetic predispositions, they do not support the clinical utility of non-validated thrombophilia testing. Future studies with prospective designs and expanded genetic panels are warranted to further delineate the biological relevance of these associations within the context of placental vascular pathology.

## Figures and Tables

**Table 1:** Classification of placental lesions according to Amsterdam Criteria and composite scoring system.

Lesion	Amsterdam Classification	Scored in Composite Score?	Histopathological Category
Placental infarction	Maternal Vascular Malperfusion (MVM)	Yes	Ischemic/vascular
Intervillous thrombosis	MVM	Yes	Thrombotic
Villous stasis (delayed maturation)	MVM	Yes	Maturation/flow-related
Stromal fibrosis	MVM/Other	Yes	Chronic ischemic
Acute atherosis (decidual vasculopathy)	MVM	Yes	Arteriopathy
Villous agglutination	Not scored in composite	No	Stagnation marker
Chorionic vasculitis/thrombosis	Fetal Vascular Malperfusion (FVM)	No	Fetal vascular
Marked villous edema	FVM/Other	No	Fetal circulatory
Chorioamnionitis	Inflammatory Lesion	No	Maternal inflammatory response
Retroplacental hematoma	MVM/Other	No	Hemorrhagic
Immature villous appearance	Delayed Maturation (Other)	No	Developmental
Fibrous villous appearance	Other/Chronic	Yes (as stromal fibrosis)	Structural/chronic

**Table 2:** Descriptive statistics of maternal and birth-related parameters in the study population.

Variable	Mean	SD	Min	Max	Description
Age	36.68	2.72	28	40	Homogeneous group of adult women.
Newborn weight (g)	2754.71	676.43	900	4000	Wide range, includes low birth weights.
Placenta weight (g)	473.36	131.33	180	826	High variability, possibly pathological.
Placenta diameter (cm)	16.98	3.17	10	30	Generally within normal range.
Placental thickness (cm)	2.34	0.93	0.0	4.5	Some extreme values noted.
Cord length (cm)	19.57	8.11	0.0	45.0	Wide variation; some very short cords.
Cord width (cm)	1.13	0.35	0.0	2.5	Normal range, but 0 may indicate error or anomaly.

**Table 3:** Effect size estimates (Cohen’s d) for selected clinical, histopathological, and genetic variables between thrombophilia and control groups. Positive values indicate higher prevalence or levels in the thrombophilia group. Bolded values reflect large effect sizes (*d* ≥ 0.8) or confidence intervals excluding zero.

Variable	Cohen’s d	95% CI (Lower–Upper)	Interpretation
PAI-1 4G/4G	1.906	1.337–2.467	Large effect
MTHFR C677T	1.846	1.281–2.402	Large effect
Factor V	0.934	0.425–1.437	Large effect
EPCR	1.060	0.546–1.569	Large effect
Factor II G20210A	0.673	0.175–1.168	Moderate–large
Villous stasis	0.647	0.150–1.140	Moderate–large
CO (cord normal)	0.830	0.327–1.330	Large effect
Retroplacental hematoma	0.678	0.180–1.172	Moderate–large
Fibrous appearance	0.457	−0.034–0.946	Medium (borderline)
Acute atherosis	0.596	0.100–1.088	Moderate
Smoking	0.551	0.057–1.042	Moderate

**Table 4:** Comparison of maternal and placental morphometric parameters between thrombophilia and control groups.

		Total		Groups				*p*
				1		2		
		Mean	SD	Mean	SD	Mean	SD	
Age		36.68	2.72	36.61	2.61	36.86	3.08	0.711
Weight of newborn		2754.71	676.43	2749.05	696.55	2770.91	630.43	0.897
Weight of placenta		473.36	131.33	474.48	135.69	470.18	120.92	0.896
Placenta diameter		16.98	3.17	16.92	3.50	17.14	1.96	0.785
Placental thickness		2.34	0.93	2.37	1.02	2.27	0.61	0.687
Umbilical cord length		19.57	8.11	19.48	8.16	19.84	8.14	0.857
Umbilical cord width		1.13	0.35	1.13	0.38	1.12	0.24	0.904
BMI		24.55	3.8	24.64	4.09	24.31	2.89	0.956
Smoking (N%)	No	53(62.4%)		35(41.2%)		18(21.2%)		0.030
	Yes	32(37.6%)		28(32.9%)		4(4.7%)		
Parity		0.48	0.63	0.43	0.61	0.64	0.66	0.153

SD = standard deviation, *p* = statistical significance.

**Table 6:** Frequency of tThrombophilia-associated genetic mutations in thrombophilia and control groups with statistical significance.

Parameters		Total		Groups				*p*
				1		2		
		Count	%	Count	%	Count	%	
Factor V	No	57	67.1	35	41.2	22	25.9	0.001 **
	Heterozygous	15	17.6	15	17.6	0	0.0	
	Homozygous	13	15.3	13	15.3	0	0.0	
Factor II G20220A	No	67	78.8	45	52.9	22	25.9	0.008 **
	Heterozygous	14	16.5	14	16.5	0	0.0	
	Homozygous	4	4.7	4	4.7	0	0.0	
MTHFR C677T	No	34	40.0	12	14.1	22	25.9	0.001 **
	Heterozygous	37	43.5	37	43.5	0	0.0	
	Homozygous	14	16.5	14	16.5	0	0.0	
MTHFR A1298C	No	55	64.7	33	38.8	22	25.9	0.001 **
	Heterozygous	19	22.4	19	22.4	0	0.0	
	Homozygous	11	12.9	11	12.9	0	0.0	
FACTOR XIII V34L	No	69	81.2	47	55.3	22	25.9	0.014 **
	Heterozygous	10	11.8	10	11.8	0	0.0	
	Homozygous	6	7.1	6	7.1	0	0.0	
PAI 1 4G4G	No	35	41.2	13	15.3	22	25.9	0.001 **
	Heterozygous	17	20.0	17	20.0	0	0.0	
	Homozygous	33	38.8	33	38.8	0	0.0	
EPCR	No	56	65.9	34	40.0	22	25.9	0.001 **
	alele a1/a3	29	34.1	29	34.1	0	0.0	

*p* = statistical significance, ** = correlation is significant at the 0.01 level (2-tailed).

**Table 7:** Prevalence and interpretation of macroscopic and microscopic placental lesions in the study cohort.

Category	Mean	Interpretation
Boiled meat appearance	0.27	Affects ~27% of cases, macroscopic sign of pathology.
Immature appearance	0.16	Seen in 16%, it could reflect delayed villous maturation.
Fibrous appearance	0.34	Around 34%, suggesting chronic stress/injury.
Placental infarction	0.38	Present in 38%—relatively frequent.
Acute placental atherosis	0.34	Common feature of maternal vascular malperfusion.
Marked villous edema	0.39	Suggestive of fetal vascular compromise.
Signs of maternal HTN	0.22	Detected in 22%, correlates with vascular pathology.
Acute hypoxia/malperfusion	0.35	Suggests fetal distress in ~35%.
Chorioamniotitis	0.18	Infection seen in ~18%.
Retroplacental hematoma	0.26	Hemorrhagic complication in ~26%.
Intervillous thrombosis	0.38	Highly prevalent feature.
Chorionic vasal thrombosis	0.13	Less frequent but severe if present.
Villous agglutinations	0.45	Almost half the placentas affected.
Villous stasis	0.51	Present in ~50%, reflecting circulatory stasis.

**Table 8:** Distribution of placental lesions in thrombophilia and control groups with sStatistical comparison.

Parameters		Total		Groups				*p*
				1		2		
		Count	%	Count	%	Count	%	
Boiled meat	No	62	72.9	43	50.6	19	22.4	0.102
	Yes	23	27.1	20	23.5	3	3.5	
Immature appearance	No	71	83.5	51	60.0	20	23.5	0.284
	Yes	14	16.5	12	14.1	2	2.4	
Fibrous appearance	No	56	65.9	38	44.7	18	21.2	0.068
	Yes	29	34.1	25	29.4	4	4.7	
Placental infarction	No	53	62.4	36	42.4	17	20.0	0.096
	Yes	32	37.6	27	31.8	5	5.9	
Acute placental atherosis	No	56	65.9	37	43.5	19	22.4	0.018 **
	Yes	29	34.1	26	30.6	3	3.5	
Marked villous edema	No	52	61.2	35	41.2	17	20.0	0.073
	Yes	33	38.8	28	32.9	5	5.9	
Signs of HTN	No	66	77.6	44	51.8	22	25.9	0.003 **
	Yes	19	22.4	19	22.4	0	0.0	
Acute hypoxia malperfusion	No	55	64.7	40	47.1	15	17.6	0.696
	Yes	30	35.3	23	27.1	7	8.2	
Chorioamniotitis	No	70	82.4	54	63.5	16	18.8	0.173
	Yes	15	17.6	9	10.6	6	7.1	
Retroplacental hematoma	No	63	74.1	42	49.4	21	24.7	0.008 **
	Yes	22	25.9	21	24.7	1	1.2	
Intervillous thrombosis	No	53	62.4	39	45.9	14	16.5	0.887
	Yes	32	37.6	24	28.2	8	9.4	
Chorionic vassal thrombosis	No	74	87.1	55	64.7	19	22.4	0.911
	Yes	11	12.9	8	9.4	3	3.5	
Villous agglutinations	No	47	55.3	31	36.5	16	18.8	0.057
	Yes	38	44.7	32	37.6	6	7.1	
Villous stasis	No	42	49.4	26	30.6	16	18.8	0.011 **
	Yes	43	50.6	37	43.5	6	7.1	

*p* = statistical significance, ** = correlation is significant at the 0.01 level (2-tailed).

**Table 9:** Prevalence and interpretation of umbilical cord structural features in the study cohort.

Feature	Mean	Interpretation
Inset (central/marginal)	0.72	Most cords are not centrally inserted.
Hypertwisted cord	0.39	Seen in ~39%—may affect fetal blood flow.
Cord coiling normal	0.72	72% have normal coiling—some variation is noted.

**Table 10:** Comparison of umbilical cord insertion, twisting, and coiling between thrombophilia and control groups.

Parameters		Total		Groups				*p*
				1		2		
		Count	%	Count	%	Count	%	
Inset	Central	38	44.7	32	37.6	6	7.1	0.281
	Eccentric	33	38.8	20	23.5	13	15.3	
	Marginal	14	16.5	11	12.9	3	3.5	
Hypertwisted	No	52	61.2	45	52.9	7	8.2	0.001 **
	Yes	33	38.8	18	21.2	15	17.6	
Cord coiling normal	Normal	24	28.2	12	14.1	12	14.1	0.001 **
	Modified	61	71.8	51	60.0	10	11.8	

*p* = statistical significance, ** = correlation is significant at the 0.01 level (2-tailed).

**Table 11:** Pearson correlation coefficients between thrombophilic mutations and maternal age, fetal, and placental measurements.

Pearson Correlation		Factor V	Factor II G20220A	MTHFR C677T	MTHFR A1298C	FACTOR XIII V34L	PAI 1 4G4G	EPCR
Age	*r*	−0.095	−0.064	0.101	−0.123	0.106	−0.108	0.054
	*p*	0.389	0.560	0.360	0.261	0.333	0.327	0.624
Weight of new born	*r*	−0.166	−0.231 *	0.159	−0.084	0.090	−0.086	0.077
	*p*	0.128	0.034	0.146	0.446	0.411	0.435	0.486
Weight of placenta	*r*	−0.191	−0.249 *	0.129	−0.003	0.030	−0.051	0.057
	*p*	0.080	0.022	0.240	0.975	0.783	0.645	0.604
Placenta diameter	*r*	−0.238 *	−0.227 *	0.118	−0.024	0.094	−0.155	0.108
	*p*	0.028	0.037	0.282	0.829	0.392	0.157	0.326
Placental thickness	*r*	−0.263 *	−0.077	0.010	−0.059	−0.115	−0.098	0.047
	*p*	0.015	0.486	0.930	0.589	0.295	0.372	0.667
	*N*	85	85	85	85	85	85	85

*r* = Pearson coefficient, *p* = statistical significance, *N* = number of patients, * = correlation is significant at the 0.05 level (2-tailed).

**Table 12:** Pearson correlation coefficients between inherited thrombophilic mutations.

Parameters		Factor V	Factor II G20220A	MTHFR C677T	MTHFR A1298C	Factor XIII V34L	PAI 1 4G4G	EPCR
Factor V	*r*	1	0.720 **	−0.030	0.116	0.229 *	0.370 **	−0.199
	*p*		0.000	0.786	0.292	0.035	0.000	0.067
Factor II G20220A	*r*	0.720 **	1	0.005	−0.050	0.088	0.259 *	−0.209
	*p*	0.000		0.961	0.651	0.424	0.017	0.055
MTHFR C677T	*r*	−0.030	0.005	1	0.200	0.205	0.378 **	0.446 **
	*p*	0.786	0.961		0.067	0.060	0.000	0.000
MTHFR A1298C	*r*	0.116	−0.050	0.200	1	0.097	0.461 **	0.244 *
	*p*	0.292	0.651	0.067		0.378	0.000	0.024
Factor XIII V34L	*r*	0.229 *	0.088	0.205	0.097	1	0.217 *	−0.108
	*p*	0.035	0.424	0.060	0.378		0.046	0.326
PAI 1 4G4G	*r*	0.370 **	0.259 *	0.378 **	0.461 **	0.217 *	1	0.130
	*p*	0.000	0.017	0.000	0.000	0.046		0.236
EPCR	*r*	−0.199	−0.209	0.446 **	0.244 *	−0.108	0.130	1
	*p*	0.067	0.055	0.000	0.024	0.326	0.236	
	*N*	85	85	85	85	85	85	85

*r* = Pearson coefficient, *p* = statistical significance, *N* = number of patients, ** = correlation is significant at the 0.01 level (2-tailed), * = correlation is significant at the 0.05 level (2-tailed).

**Table 15:** Multiple linear regression predicting histologic composite score, adjusted for smoking, BMI, gestational age, and parity.

Predictor	β (95% CI)	*p*-Value
Thrombophilia	+2.3 (1.1–3.5)	0.001
Smoking	+0.9 (–0.3–2.1)	0.14
BMI	+0.05 (–0.02–0.12)	0.18
Gestational age	–0.2 (–0.4–0.0)	0.045
Parity	–0.1 (–0.3–0.1)	0.25

β = regression coefficient; 95% CI = confidence interval.

## Data Availability

All the data processed in this article are part of the research for a doctoral thesis, being archived in the aesthetic medical office where the interventions were performed.

## Supplementary Materials

The following supporting information can be downloaded at: https://www.mdpi.com/article/10.3390/cimb47080612/s1.

## References

[1] Khan S., Dickerman J.D. (2006). Hereditary thrombophilia. Thromb. J..

[2] Murvai V.R., Radu C.-M., Galiș R., Ghitea T.C., Tătaru-Copos A.-F., Vesa A.-A., Huniadi A. (2025). The relationship between thrombophilia and modifications in first-trimester prenatal screening markers. Medicina.

[3] Murvai V.R., Galiș R., Panaitescu A., Radu C.M., Ghitea T.C., Trif P., Onița-Avram M., Vesa A.A., Huniadi A. (2025). Antiphospholipid syndrome in pregnancy: A comprehensive literature review. BMC Pregnancy Childbirth.

[4] Goodman C.S., Coulam C.B., Jeyendran R.S., Acosta V.A., Roussev R. (2006). Which thrombophilic gene mutations are risk factors for recurrent pregnancy loss?. Am. J. Reprod. Immunol..

[5] Samfireag M., Potre C., Potre O., Tudor R., Hoinoiu T., Anghel A. (2022). Approach to thrombophilia in pregnancy—A narrative review. Medicina.

[6] Dyer L.A., Rugonyi S. (2021). Fetal blood flow and genetic mutations in conotruncal congenital heart disease. J. Cardiovasc. Dev. Dis..

[7] Wintermark P., Boyd T., Parast M.M., Van Marter L.J., Warfield S.K., Robertson R.L., Ringer S.A. (2010). Fetal placental thrombosis and neonatal implications. Am. J. Perinatol..

[8] Cornish E.F., McDonnell T., Williams D.J. (2022). Chronic inflammatory placental disorders associated with recurrent adverse pregnancy outcome. Front. Immunol..

[9] Kim Y.M., Chaemsaithong P., Romero R., Shaman M., Kim C.J., Kim J.S., Qureshi F., Jacques S.M., Ahmed A.I., Chaiworapongsa T. (2015). Placental lesions associated with acute atherosis. J. Matern. Fetal Neonatal Med..

[10] Menter T., Bruder E., Hösli I., Lapaire O., Raio L., Schneider H., Höller S., Hentschel R., Brandt S., Bode P. (2024). Pathologic findings of the placenta and clinical implications—Recommendations for placental examination. Swiss Med. Wkly..

[11] Kalluru P.K.R., Kalluru H.R., Allagadda T.R., Talur M., Gonepogu M.C., Gupta S. (2024). Abnormal umbilical cord coiling and association with pregnancy factors. J. Turk. Ger. Gynecol. Assoc..

[12] Todtenhaupt P., Kuipers T.B., Dijkstra K.L., Voortman L.M., Franken L.A., Spekman J.A., Jonkman T.H., Groene S.G., Roest A.A., Haak M.C. (2024). Twisting the theory on the origin of human umbilical cord coiling featuring monozygotic twins. Life Sci. Alliance.

[13] Burlacu D., Burlacu A., Călburean P., Szabo B., Mezei T. (2025). Correlation of the umbilical cord coiling pattern and fetal outcome: A single-center observational analytical study. Placenta.

[14] Saw S.N., Dai Y., Yap C.H. (2021). A review of biomechanics analysis of the umbilical—Placenta system with regards to diseases. Front. Physiol..

[15] Deloughery T.G., Hunt B.J., Barnes G.D., Connors J.M., Ay C., Barco S., Castellucci L., Cesarman-Maus G., Paula E.V.D., Dumantepe M. (2022). A call to action: Mthfr polymorphisms should not be a part of inherited thrombophilia testing. Res. Pract. Thromb. Haemost..

[16] Many A., Schreiber L., Rosner S., Lessing J.B., Eldor A., Kupferminc M.J. (2001). Pathologic features of the placenta in women with severe pregnancy complications and thrombophilia. Obs. Gynecol..

[17] Ernst L. (2018). Maternal vascular malperfusion of the placental bed. APMIS.

[18] Nogueira R., Soares Nogueira F., Ahmad R. (2025). Placental malperfusion in maternal diseases. Lupus—Diagnostics and Developments.

[19] Beeksma F., Erwich J.J., Khong T. (2012). Placental fetal vascular thrombosis lesions and maternal thrombophilia. Pathology.

[20] Redline R.W., Ravishankar S., Bagby C.M., Saab S.T., Zarei S. (2021). Four major patterns of placental injury: A stepwise guide for understanding and implementing the 2016 Amsterdam consensus. Mod. Pathol..

[21] Kim R.J., Becker R.C. (2003). Association between factor v leiden, prothrombin g20210a, and methylenetetrahydrofolate reductase c677t mutations and events of the arterial circulatory system: A meta-analysis of published studies. Am. Heart J..

[22] Padda J., Khalid K., Mohan A., Pokhriyal S., Batra N., Hitawala G., Cooper A.C., Jean-Charles G. (2021). Factor v leiden g1691a and prothrombin gene g20210a mutations on pregnancy outcome. Cureus.

[23] Kinzler W.L., Prasad V., Ananth C.V. (2009). The effect of maternal thrombophilia on placental abruption: Histologic correlates. J. Matern. Fetal Neonatal Med..

[24] Said J., Tranquilli A.L. (2011). The impact of inherited thrombophilia on placental haemostasis and adverse pregnancy outcomes. Thrombophilia.

[25] Gris J.-C. (2009). Thrombophilia and pregnancy loss: Cause or association. Thromb. Res..

[26] Dimitriadis E., Menkhorst E., Saito S., Kutteh W.H., Brosens J.J. (2020). Recurrent pregnancy loss. Nat. Rev. Dis. Primers.

[27] Paternoster D.M., Surico N., Riboni F., Gambaro C., Girolami A., Milani M., Nicolini U. (2007). Thrombophilias in pregnancy and their role in preeclampsia. Expert. Rev. Obstet. Gynecol..

[28] Faye-Petersen O. (2008). The placenta in preterm birth. J. Clin. Pathol..

[29] Kupferminc M.J., Peri H., Zwang E., Yaron Y., Wolman I., Eldor A. (2000). High prevalence of the prothrombin gene mutation in women with intrauterine growth retardation, abruptio placentae and second trimester loss. Acta Obstet. Gynecol. Scand..

[30] Pasta L., Pasta F., D’Amico M. (2016). Pai-1 4g-4g, mthfr 677tt, v leiden 506q, and prothrombin 20210a in splanchnic vein thrombosis: Analysis of individual patient data from three prospective studies. J. Clin. Exp. Hepatol..

[31] Pop T.R., Vesa Ş.C., Trifa A.P., Crişan S., Buzoianu A.D. (2014). Pai-1 4g/5g and mthfr c677t polymorphisms increased the accuracy of two prediction scores for the risk of acute lower extremity deep vein thrombosis. Rom. J. Morphol. Embryol..

[32] Oztuzcu S., Ergun S., Ulaşlı M., Nacarkahya G., Iğci Y.Z., Iğci M., Bayraktar R., Tamer A., Cakmak E.A., Arslan A. (2014). Evaluation of factor v g1691a, prothrombin g20210a, factor xiii v34l, mthfr a1298c, mthfr c677t and pai-1 4g/5g genotype frequencies of patients subjected to cardiovascular disease (cvd) panel in south-east region of Turkey. Mol. Biol. Rep..

[33] James A.H., Brancazio L.R., Ortel T.L. (2005). Thrombosis, thrombophilia, and thromboprophylaxis in pregnancy. Heart Dis..

[34] Sadr M., Naemi B., Tafrihi M., Esfehani R.J. (2025). Genetic variants in mthfr and pai-1 genes and their influence on miscarriage risk in the Iranian population. Hum. Gene.

[35] Sokol Karadjole V., D’Amato A., Milošević M., Herman M., Mikuš M., Laganà A.S., Chiantera V., Etrusco A. (2024). Impact of thrombophilic polymorphisms in antenatal women on perinatal health: A single-center prospective study. J. Pers. Med..

