# Supplementary material for: Prothrombotic Genetic Mutations Are Associated with Sub-Clinical Placental Vascular Lesions: A Histopathological and Morphometric Study
Source: Curr Issues Mol Biol. 2025 Aug 4;47(8):612. doi: 10.3390/cimb47080612 (PMC12384614; doi:10.3390/cimb47080612)
Supplement: Supplementary file 1 [file cimb-47-00612-s001.zip › cimb-3764824-supplementary.pdf]

A 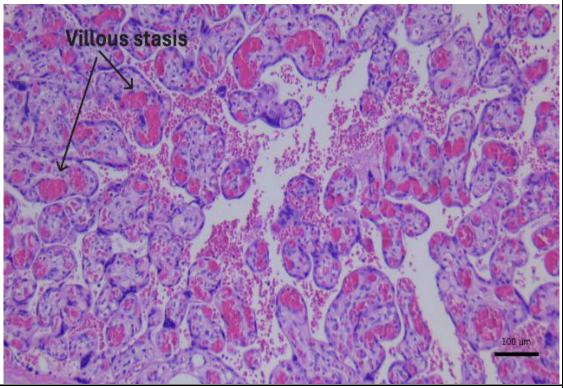 Villous stasis<br>100 µm	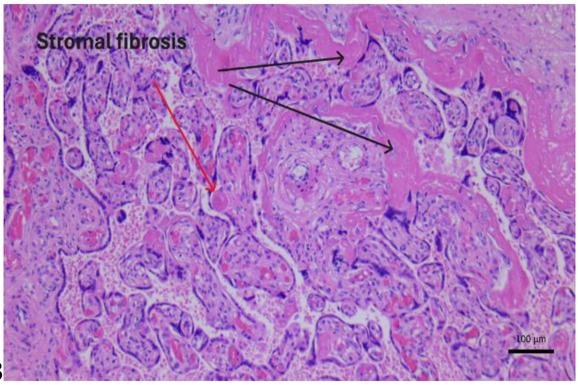 Stromal fibrosis<br>100 µm B
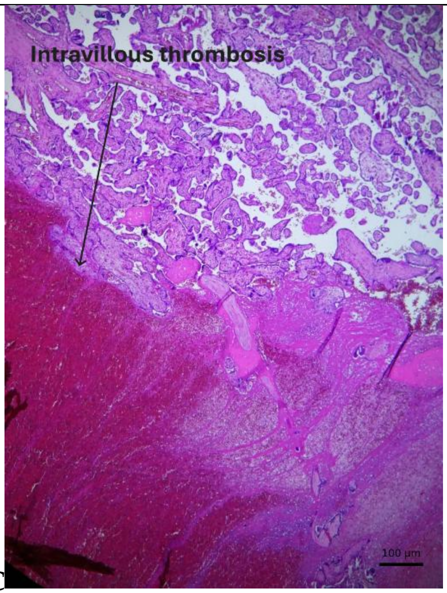 Intravillous thrombosis<br>100 µm C	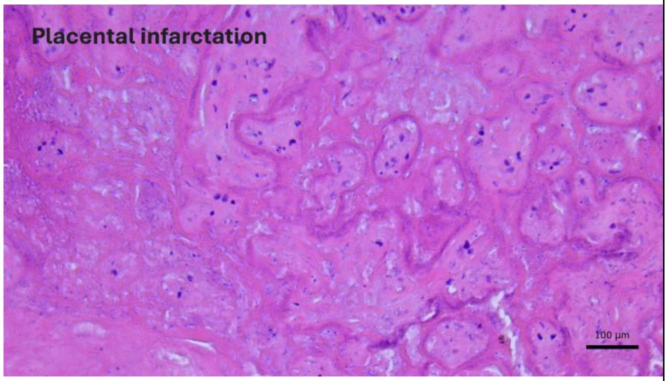 Placental infarctation<br>100 µm D
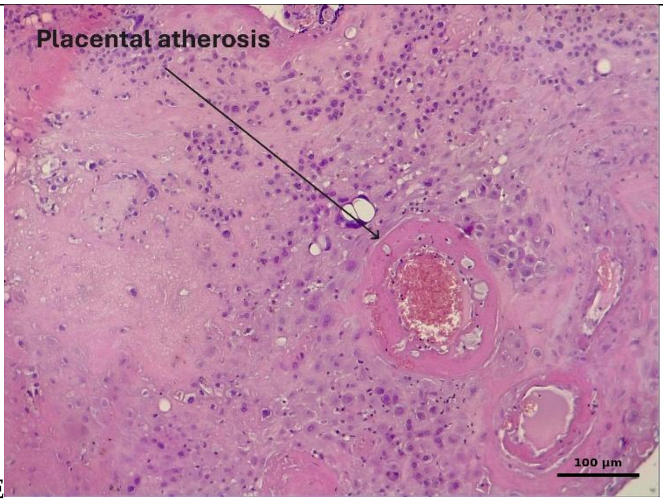 Placental atherosis<br>100 µm E	

Supplementary Figure 1. Representative histologic images illustrating scoring criteria for placental lesions. Separate panels are shown for (A) villous stasis, (B) stromal fibrosis, (C) intravillous thrombosis, (D) placental infarction, and (E) acute atherosis. For each feature, images include examples with and without the lesion (control) to demonstrate the morphological criteria used in scoring.
